# Effects of RNAi of Ribophorin 1, Eukaryotic translation initiation factor 2 beta and Peptidylprolyl isomerase A in *Acyrthosiphon pisum*

**Published:** 2026-05-20

**Authors:** Brandon Kennemer, James R Balthazor

**Affiliations:** 1 Department of Biological Sciences, Fort Hays State University, Hays, KS United States of America; 2 Department of Chemistry, Fort Hays State University, Hays,KS United States of America

**Keywords:** dsRNA, RNAi, Pest mitigation, UPR

## Abstract

Three genes found in the Unfolded protein response, Ribophorin 1, Eukaryotic translation initiation factor 2 beta and peptidylprolyl isomerase a are thought to be potential targets for RNA interference (RNAi) in *Acyrthosiphon pisum*. Ribophorin 1 (RPN1) is a transmembrane glycoprotein that assists in anchoring ribosomes to the rough endoplasmic reticulum membrane. It acts as a substrate specific chaperone. It facilitates N-glycosylation by delivering newly synthesized proteins to the Oligosaccharyl Transferase (OST) complex. Eukaryotic translation Initiation Factor 2 Beta (eIF2B) is a key component of the eIF2 heterotrimer that facilitates protein synthesis initiation by binding GTP and recruiting a specific transfer RNA to the 40S ribosome. The RNA it recruits is tRNAiMet, which delivers the first methionine to the ribosome to start protein synthesis. eIF2B is part of both the Unfolded Protein Response (UPR) and the Integrated Stress Response (ISR). Peptidylprolyl Isomerase A (PPIA) is also known as Cyclophilin A (CypA). CypA is a molecular chaperone that catalyzes the cis-trans isomerization of peptide bonds, which facilitates protein folding and maturation during stressful conditions. Previous studies confirm that RNAi can affect the lifespan and fecundity of pea aphids. The objective of this study was to determine whether the selected genes would also affect the lifespan of pea aphids. Decreasing concentrations of double-stranded Ribo Nucleic Acid (dsRNA) were fed to the aphids to test the effects of each chosen gene. The experiment’s objective is to identify the effects of each dsRNA knockdown on the aphids. Higher concentrations had a greater effect on decreasing aphid survival. RPN1 and CypA reduced aphid survival only at the highest concentration tested. eIF2B showed a greater effect on aphid survival at lower concentrations than the other genes tested. It was effective at decreasing survival at a concentration of 100 ng/mL. The result of this study agrees with previous studies that aphid survival can be affected by the introduction of dsRNAs.

## INTRODUCTION

Pests pose a serious threat to farmers’ crops, causing an estimated $120 billion in damage annually [1]. Among these, insect pests are a primary concern for research groups, as many species are developing resistance to traditional control methods. While some insects only cause cosmetic damage, those that harm plant growth represent a significant problem for farmers, who are eager for substances that can reduce pest populations and increase crop yields.

Traditionally, pest control relies on spraying insecticides directly onto plants. The most commonly used classes of insecticides include organophosphates, pyrethroids and carbamates [2]. However, these chemicals can have harmful effects on humans and non-target organisms. Organophosphates, the most toxic class still in use, have seen reduced application since 2001 following the discovery of their fatal effects on non-target species [3]. Despite them still being in use, many aphid species have developed immunity to at least one type of insecticide, limiting the effectiveness of chemical control [4].

Aphid infestations are particularly damaging to crops such as wheat in the northwest United States, where annual losses reach $120 million [5]. Aphids feed by inserting their proboscis into plant leaves or stems and siphoning sap, which stunts plant growth and causes discoloration. When feeding, aphids first secrete a gel-like saliva to seal the puncture site, followed by a watery saliva containing effector proteins that counter the plant’s defenses [6,7]. To obtain sufficient nutrients, aphids consume large amounts of sap, excreting the excess as honeydew. This honeydew attracts ants, which act as bodyguards in exchange for the sugary secretion, protecting the colony from predators. While this mutualistic relationship allows aphid populations to grow, the honeydew also promotes fungal growth, further reducing crop yield [8].

Another impact on crops stems from the exponential population growth in offspring, which is due to their ability to reproduce by parthenogenesis. Parthenogenesis is asexual reproduction, where the offspring are clones of the mother. Aphids are born gravid, so that a new aphid can produce multiple clones rapidly. An aphid infestation can occur in a week. An infestation starts when winged aphids travel to a new location and start a new colony on the plants. The aphids will start feeding on the crops. The original aphids will feed until they are big enough to reproduce. A single aphid is capable of producing 40 to 60 offspring. Each one of those offspring is already born gravid.

Aphids have become increasingly resistant to traditional pest control methods. Insecticides are no longer viable control agents at the levels previously used. At high insecticide levels, aphid infestations can still be controlled. A study showed that at least 20 aphid species have become immune to at least one insecticide [9]. Spraying more insecticides affects the environment and the development of new resistances. The growing issue with insecticide resistance is that if farmers notice the insecticide is not working, they might spray more. The extra insecticide spray could run into a stream, leading to more insects developing resistances [10]. Insecticide runoff could damage trees, plants and other wildlife exposed to it. If RNAi works correctly to control pests, there will be no harmful runoff or off-target effects.

Ribonucleic Acid interference (RNAi) is a method of silencing target genes. Silencing occurs in two steps once dsRNA has been introduced into the cell. The long-stranded dsRNA is processed by an RNase enzyme (dicer). Dicer cleaves long-stranded dsRNA into small (20–22 nucleotide long) interfering RNAs small interfering Ribo Nucleic Acid (siRNA). The siRNA directs the cleavage of homologous mRNA in the cell by an RNA-inducing silencing complex.

RNAi was first discovered in 1998, when authors identified a mechanism that degrades the mRNA of a specific gene. RNAi was used in *Caenorhabditis elegans* to manipulate gene expression [11]. Since the discovery of RNAi, many different uses have been discovered. RNAi has been used in drug development and to treat gene mutations [12,13]. RNAi was shown to silence mutant Huntingtin (HTT) proteins in a laboratory setting by inhibiting Huntington’s disease in mice [14].

The dsRNA is ingested orally, so a safe way was needed to deliver the dsRNA to the aphid. Branched Amphiphilic Peptide Capsules (BAPCs) are used as a transport capsule. BAPCs are critical for delivering dsRNA to the target area because they protect it from degradation. BAPC encapsulates the dsRNAs and protects them from digestive enzymes, nucleases, and detergents. The BAPCs also help cross barriers to aid in the delivery of dsRNAs [15].

The genes for this study were selected from the unfolded protein response. The unfolded protein response is a stress management system activated in the endoplasmic reticulum when misfolded proteins accumulate. The UPR is inactive under normal conditions but becomes active when the cell is stressed. Three different pathways activate the UPR. Inositol-Requiring Enzyme 1 (IRE1), Protein kinase RNA-like ER Kinase (PERK) and Activating Transcription Factor 6 (ATF6) all play vital roles in restoring cellular homeostasis [16]. To activate the UPR, the inactive IRE1, PERK and ATF6 proteins bound to glucose response protein 78 (GRP78) encounter unfolded protein and GRP78 is released from the proteins, leading to activation.

The three genes selected for this study are Ribophorin 1 (RPN1), Eukaryotic translation initiation factor 2 beta (eIF2B) and Peptidylprolyl isomerase A (PPIA), also known as Cyclophilin A (CypA). The genes were selected from a list of identified gene homologs [17].

RPN1 is part of the Oligosaccharyl Transferase (OST) complex. RPN1 is one of the eight subunits of the OST complex that mediates the N-glycosylation of nascent polypeptide chains entering the endoplasmic reticulum. In previous studies, RPN1 has been shown to enhance N-glycosylation of membrane proteins significantly, but it is not essential for N-glycosylation. The model proposed by Wilson in 2007 on OST function states that Ribophorin 1 acts as a chaperone to promote N-glycosylation by the catalytic STT3 subunits [18,19]. RPN1 is located on chromosome 3 in humans. A disease associated with RPN1 is Acute Myeloid Lymphoma (AML). Abnormalities on chromosome 3 that rearrange RPN1 close to EVI1 lead to Acute Myeloid Lymphoma (AML). When RPN1 is close to EVI1, RPN1 enhances EVI1 expression, leading to increased cell proliferation and decreased differentiation [20].

eIF2 is a G protein critical to translation. eIF2B is highly regulated in the integrated stress response by the phosphorylation of eIF2α [21]. eIF2B is a key component of the eIF2 heterotrimer that facilitates protein synthesis initiation by binding GTP and recruits a specific transfer RNA to the 40S ribosome. The RNA it recruits is tRNAiMet, which delivers the first methionine to the ribosome to start protein synthesis. eIF2B is part of both the Unfolded Protein Response (UPR) and the Integrated Stress Response (ISR).

eIF2B regulated protein synthesis by recycling inactive eIF2-GDP to active eIF2-GDP. The activation of the eIF2-GDP allows translation initiation. In the UPR when unfolded proteins are sensed PERK is activated. PERK phosphorylates another subunit of the eIF2 complex. The subunit phosphorylated and activated in eIF2a. When eIF2a is activated, it acts as a competitive inhibitor, binding strongly to eIF2B and stopping protein synthesis [22].

Peptidylprolyl Isomerase A (PPIA), also known as Cyclophilin A (CypA), is a molecular chaperone that catalyzes the cis-trans isomerization of peptide bonds by facilitating protein folding and maturation, particularly under stressful conditions. In addition to accelerating protein folding, CypA can be secreted from cells, where it functions as an autocrine or paracrine signaling molecule [23,24]. National Center for Biotechnology Information (NCBI) It is released in response to stimuli such as hypoxia, infection and oxidative stress. Extracellular CypA has been shown to stimulate pro-inflammatory signaling in endothelial cells and vascular smooth muscle cells. Also, CypA has been involved in a wide range of human diseases, including cancer, neurodegenerative disorders and viral infections. Notably, studies consistently report that CypA expression is upregulated in these disease contexts [25].

Previous studies have shown that RNAi can reduce aphid lifespan. In studies, aphid survival was shown to have been affected by the treatments [26,27]. In an unpublished study negative controls have been shown to have no effect on aphid survival.

The objective of this study is to determine whether the RNAi of the selected genes negatively affect aphid survival. If a gene shows a significant result at the lowest concentration, it would be selected for further studies.

## MATERIALS AND METHODS

### Insect Habitat

The aphids were kept in two grow tents. The aphids fed on *Vicia faba*, also known as broad bean plants. The broad bean plants were grown in 30-by-40-centimeter self-watering trays. The aphids are not kept at a tracked temperature; they were held at the building’s temperature. The habitats were kept on a constant light cycle simulating a 12-hour day and a 12-hour night.

### Gene Picking

The three genes were selected from a list of genes elucidated from “Tit6”. To identify genes in aphids, known human genes were compared with aphid genomes using BLAST and the results were verified in geneious. The homolog identified in *A. pisum* was used as the aphid gene sequence.

### Primer Selection

To find primers for each gene, GenBank was used. First, the NCBI Reference Sequence number was found by searching the gene name in the nucleotide database. Select one of the options after the search. Use the pick primers option to search for primers. Using taxid 7029 for *A. pisum*, search for pea aphid primers, to reduce PCR error, the product size of each primer pair was to be between 250 and 450 bp as shown in Table 1–3.

### Selected Primers

The dsRNA primers for each gene contained the T7 promoter sequence TAATACGACTCACTATAGGG. The T7 promoter sequence was added to the beginning of each primer. The primer pairs for the selected genes are located in of the Appendix (Table 4).

### RNA Isolation Steps

Prepare the working area by cleaning with RNase Away reagent. Ten adult pea aphids were collected in a 1.5 RNase-free micro centrifuge tube and homogenized in 1.0 mL of Total RNA Isolation (TRIzol) Reagent with a rotating pestle motor. The sample was centrifuged at 12000 × g and 4°C for 10 minutes. Decant the centrifuged sample into a new micro centrifuge tube and add 240 μL of chloroform. Then the sample was centrifuged in the same conditions as before. Remove the upper aqueous layer and add it to a new micro centrifuge tube. Then 500 μL of cold isopropyl alcohol was added. Let the mixture sit in an ice bath to facilitate RNA precipitation. The sample was centrifuged at 15000 × g at 4°C for 12 minutes. The pellet was then decanted and 100 μL of ethanol was used to wash it. The ethanol was decanted, and 50 μL of RNAse-free water was added. The sample was then incubated at 37°C for 1 minute. A Nanodrop was used to assess the quality of the extracted RNA. The ratio of RNA to DNA was measured at wavelengths between 260 nm and 280 nm. A quality result is a ratio above 1.9.

### cDNA Synthesis

The RNA isolated in the first step is used to synthesize stands for complementary DNA cDNA with an Applied bio systems high-capacity RNA to cDNA Kit. The quantity and quality of the cDNA will be obtained by UV absorbance. cDNA is stored at −80 degrees Celsius until dsRNA is ready to be synthesized. PCR settings were 42 degrees Celsius for 20 minutes, then 85 degrees Celsius for 6 minutes, then 4 degrees Celsius for 1 minute.

### Synthesis of dsRNAs

The cDNA synthesized and frozen in the last step is used to synthesize dsRNA. An Invitrogen 5X MEGAscript T7 kit was used to synthesize the dsRNA. 1 μL of cDNA and 2 μL of primers were used to synthesize the dsRNA. The manufacturer’s instructions were used to run the PCR with the cDNA and primers. The quality of the synthesized dsRNA was measured using UV absorbance. The measured dsRNA is stored at −80 degrees Celsius until the feeding studies are ready to begin. Polymerase Chain Reaction (PCR) settings were 90 degrees Celsius, the 95 degrees Celsius, then 55 degrees Celsius, then 72 degrees Celsius all steps were repeated 40 times to maximize dsRNA concentration.

### Modified diet to introduce dsRNAs

A specific modified diet is needed to deliver dsRNA to the aphids. The dsRNA is included in the Akey-Beck diet given to the aphids. The Akey-Beck diet is listed in of the appendix (Table 5). BAPCs were prepared and the dsRNA was incorporated into the complexes to be fed to the aphids.

### Feeding Study

A feeding study was done to test the effectiveness of the dsRNA. There was a control and 3 test groups to evaluate the effectiveness of the gene knockdowns. The test aphids were fed a modified diet [28]. For each selected gene, multiple test groups were taken and fed decreasing concentrations of dsRNA in the modified diet. The modified diet consisted of a Akey-Beck diet, 100 μL of BAPC and enough dsRNA to reach each concentration. The aphids were fed a 1 μg/mL dsRNA diet, a 100 ng/mL dsRNA diet and a 10 ng/mL dsRNA diet. 50 adult pea aphids were placed into a petri dish. A piece of parafilm was stretched very tightly to cover the top. 1 mL of the diet is pipetted onto the parafilm. Once the diet had been placed on, another piece of parafilm was stretched on top. The bottom parafilm layer acted as an artificial leaf that the aphids could pierce with their proboscis. The aphids were observed every 6 hours, with the number of dead and alive aphids recorded. The aphids were observed until all aphids in every treatment have died.

### Statistical Analysis

The results were interpreted using a Log-rank test. The specific type of log-rank test was a mentel-cox test. The significance was 0.05 for each test. If the p-value for the treatment was lower than 0.05, the treatment showed a significant difference from the control.

## RESULTS

Each feeding study was analyzed using a mentel-cox test. Data collected from each trial are located in the appendix.

**Figure 1:**
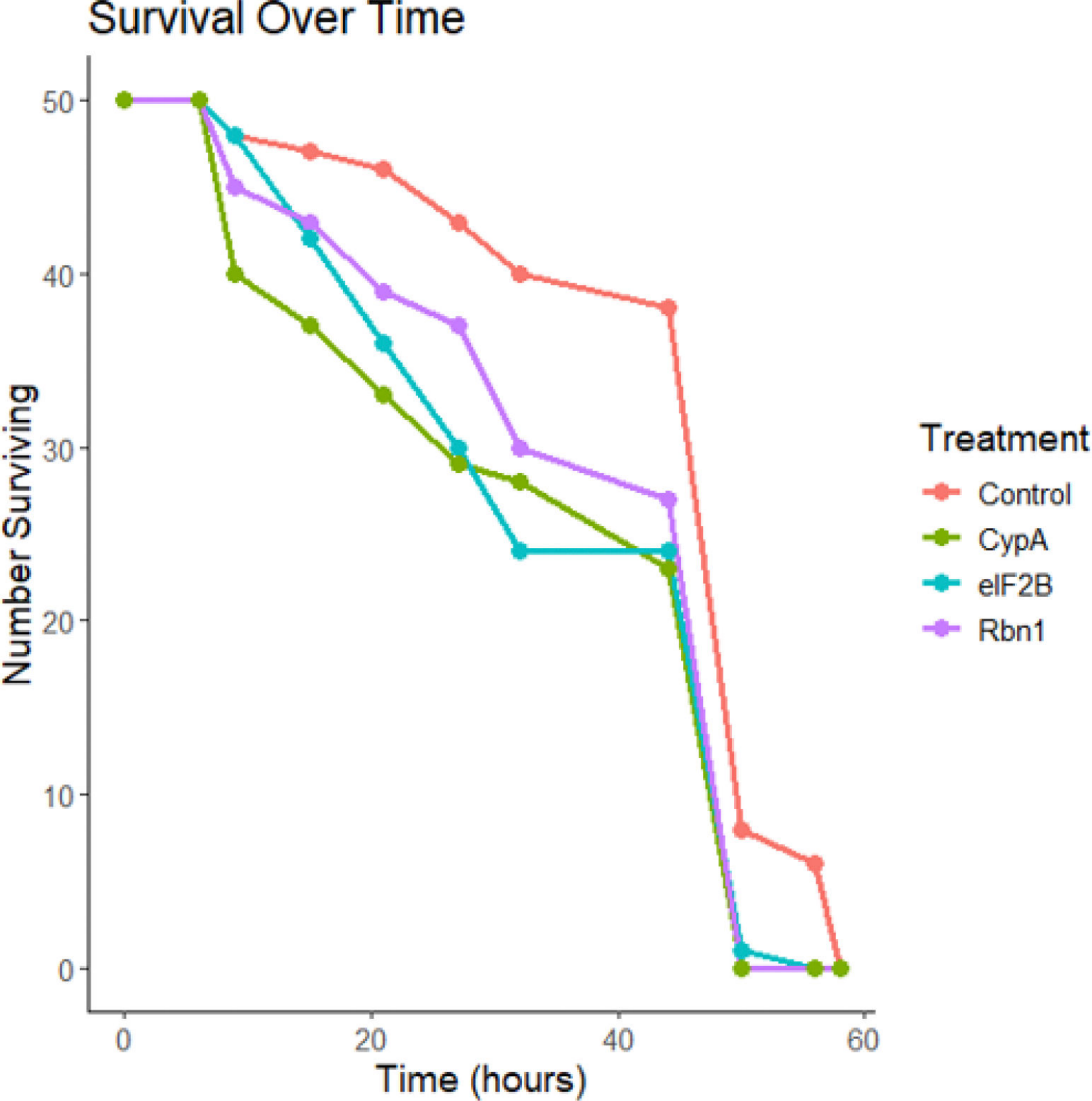
Survival of pea aphids fed 1 ug/mL of dsRNA.

**Figure 2:**
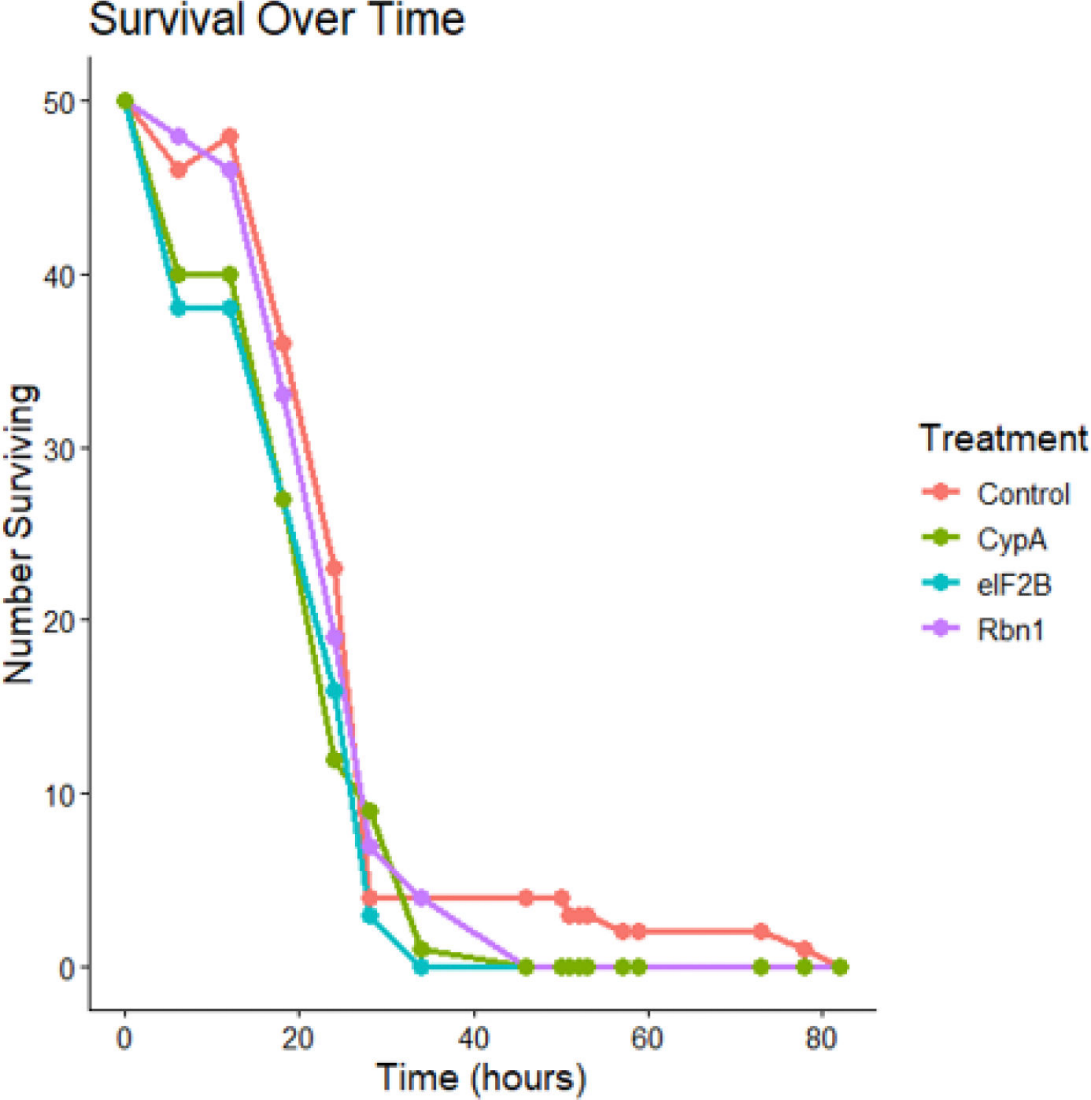
Survival of pea aphids fed 100 ng/mL of dsRNA.

**Figure 3:**
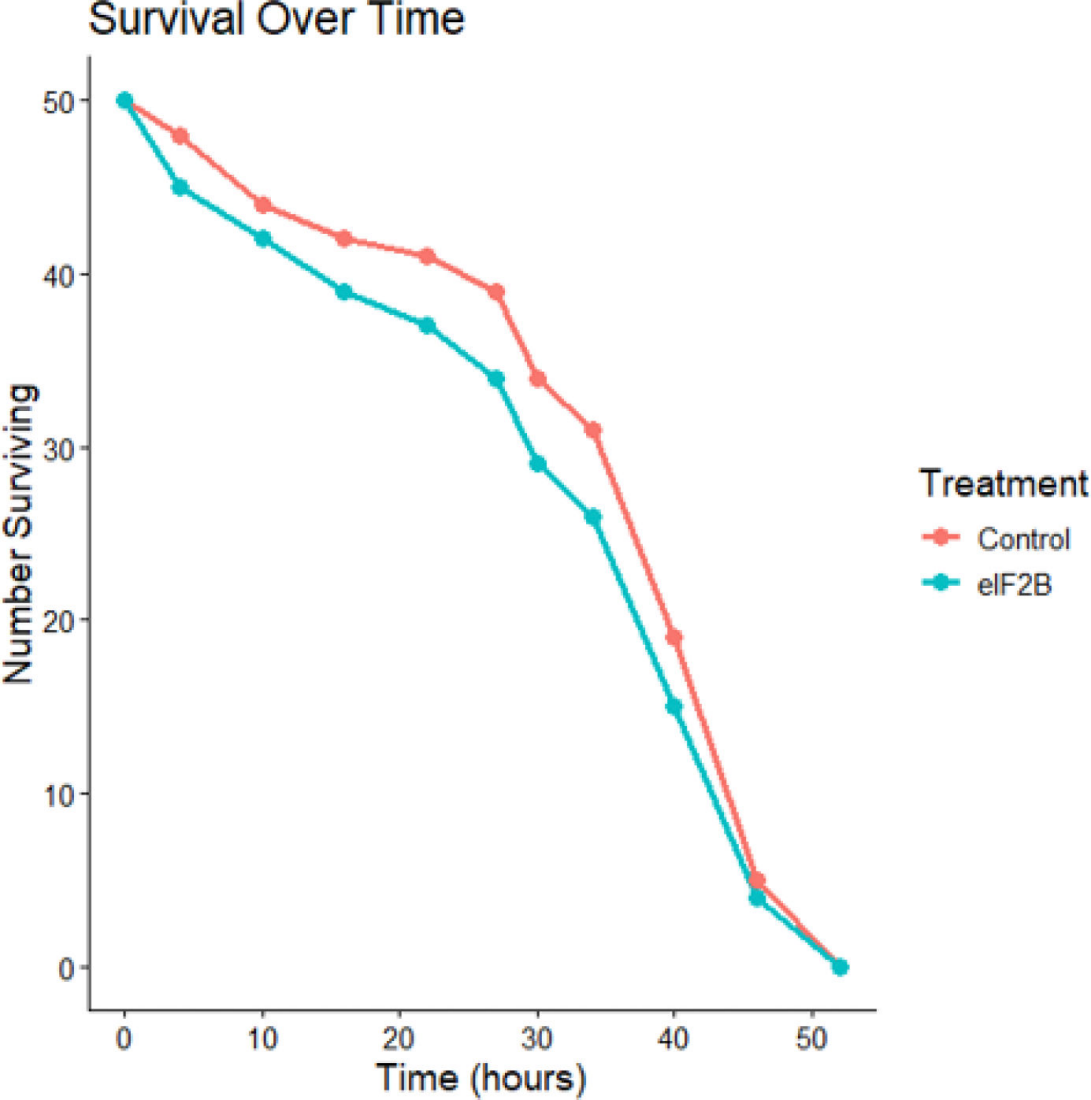
Survival of pea aphids during a 10 ng/mL dsRNA feeding study.

- **1 ug/mL feeding study:** All treatments showed a significant decrease in aphid survival. The p-value was less than 0.05 in all treatments (Figure 1). Table 1 contains the observations gathered from the feeding study.
- **100 ng/mL feeding study:** eIF2B was the only treatment that showed a significant difference. The p-value was less than 0.05. CypA showed a minor effect on aphid survival. The p-value was 0.085, indicating a minor difference from the control (Figure 2). Only eIF2B continued to a lower-concentration feeding study, since it showed a significant difference. Table 2 contains the observations of the feeding study.
- **10 ng/mL feeding study:** eIF2B was not significant at this dsRNA concentration. The p-value was 0.313 (Figure 3). Table 3 contains the observations collected from the feeding study.

## DISCUSSION

Previous studies on RNAi in aphids have demonstrated that targeting genes involved in stress response and protein folding pathways can significantly reduce aphid lifespan. Specifically, genes associated with the Unfolded Protein Response (UPR), including *PERK*, *ATF4*, *IRE1*, *HSPA1L* and *HSP90B1*, have been investigated. Among these, *ATF4* was identified as the most effective target, as its knockdown resulted in substantial reductions in aphid survival even at low dsRNA concentrations. Although all genes tested contributed to decreased lifespan at higher concentrations, the heightened sensitivity observed with *ATF4* suggests that it plays a central regulatory role in maintaining cellular homeostasis under stress conditions [29–31].

**Figure 4:**
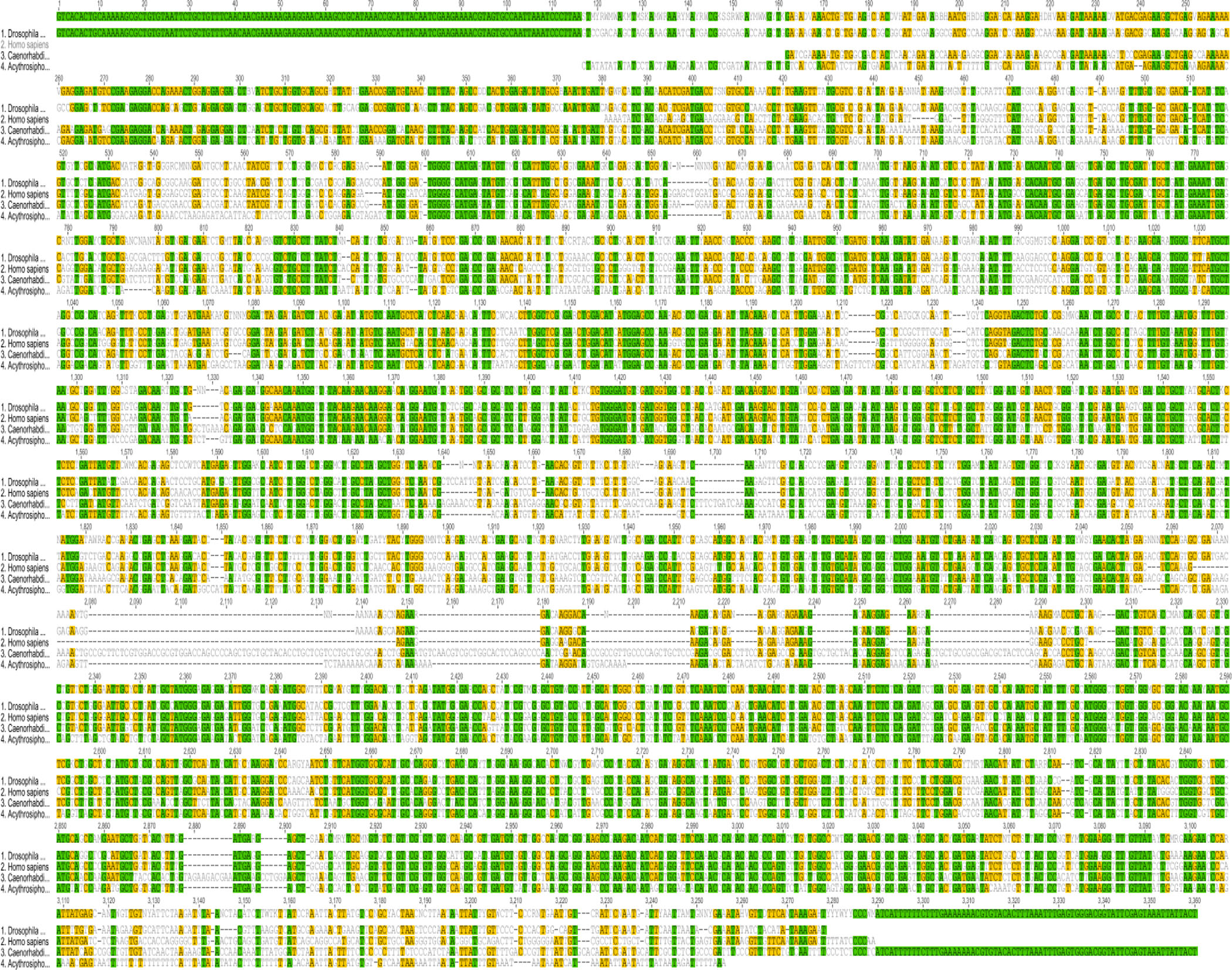
Gene sequence alignment of RPN1.

**Figure 5:**
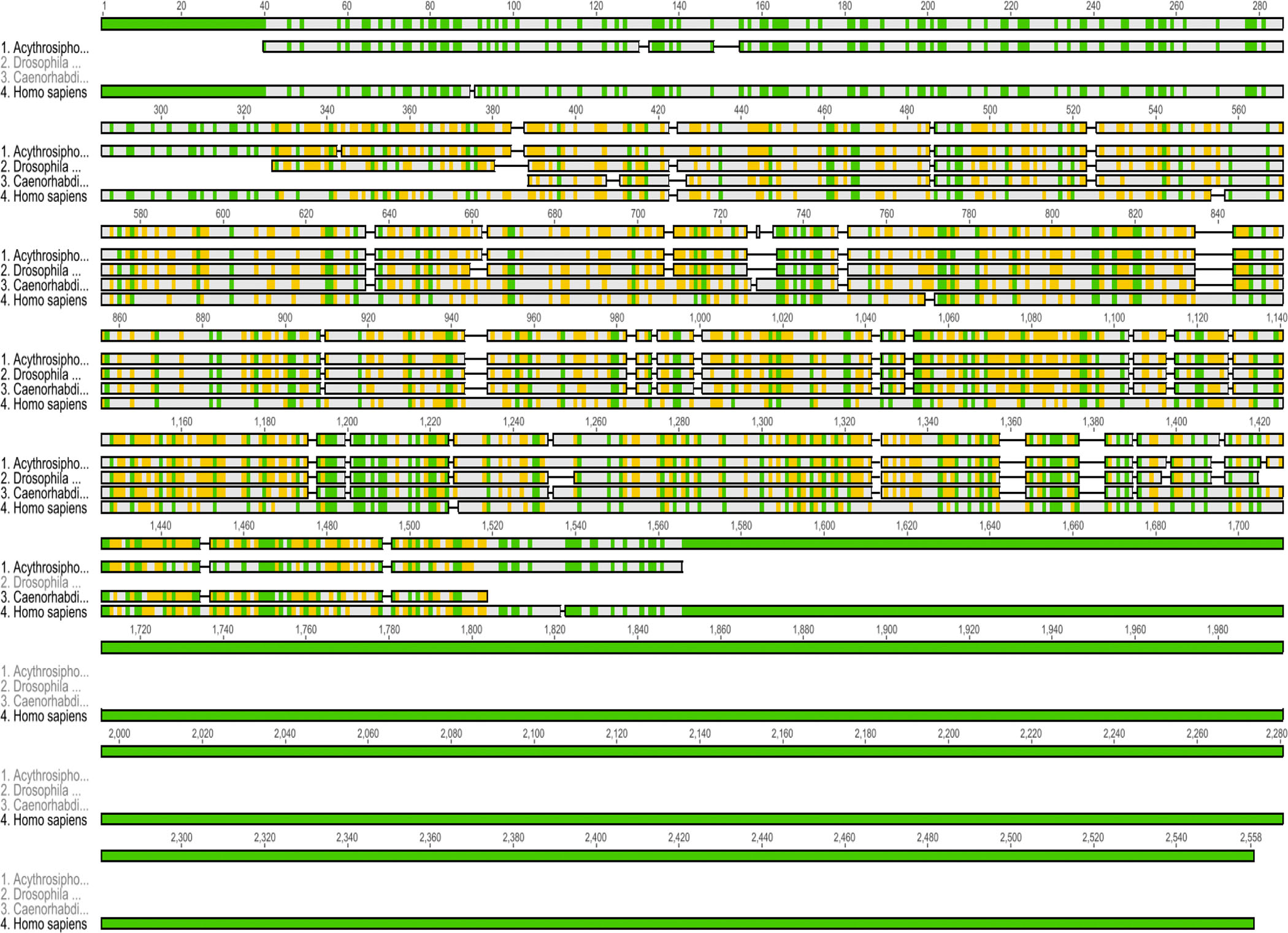
Gene sequence alignment of eIF2B.

**Figure 6:**
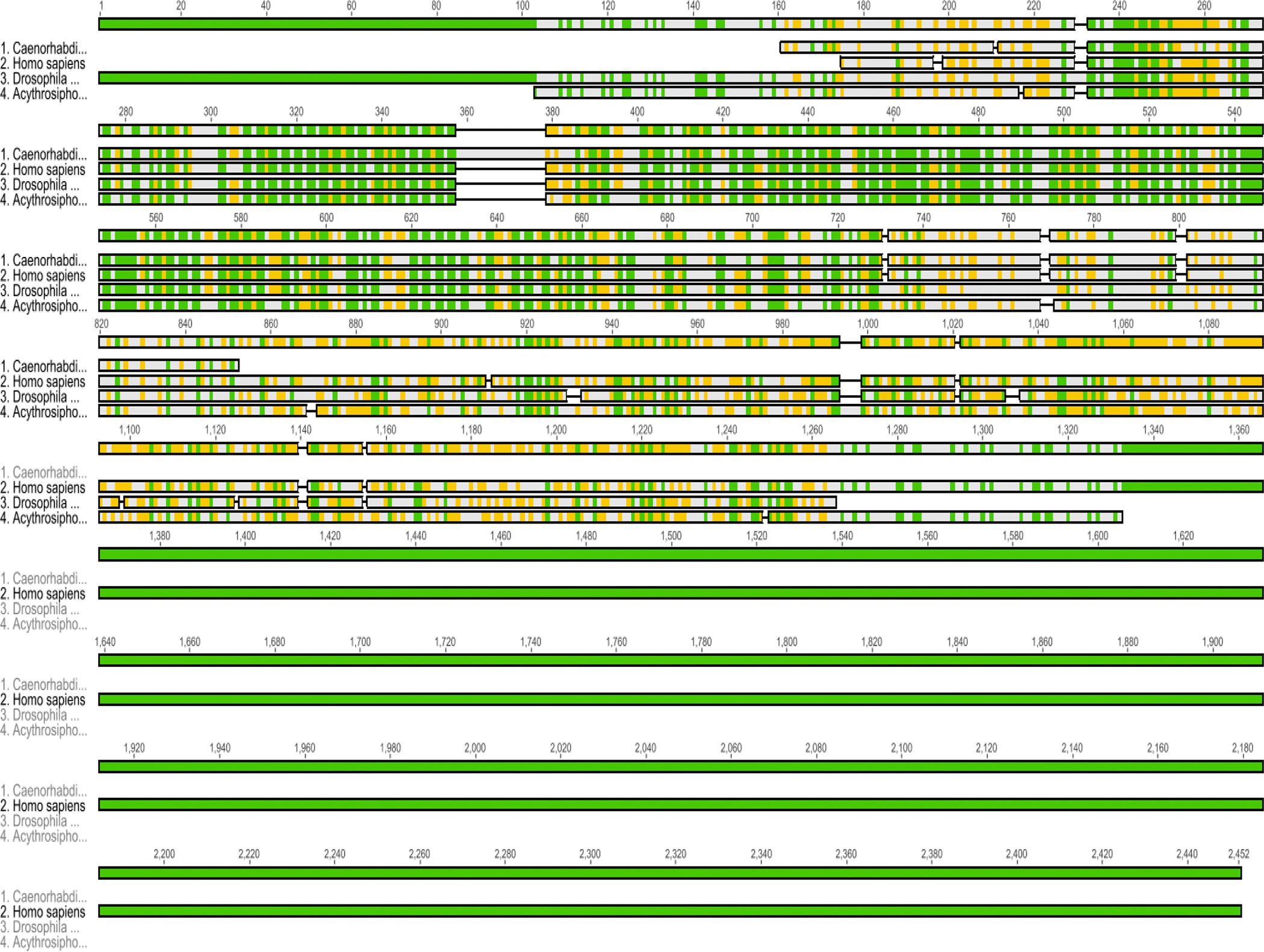
Gene sequence alignment of CypA.

The findings of this study are consistent with these previous studies, further supporting the effectiveness of RNAi-mediated gene knockdown as a strategy to reduce aphid survival. As observed previously, increased dsRNA concentrations correlated with decreased survival rates [32]. However, not all genes evaluated in this study produced significant results at lower concentrations. Specifically, RPN1 and CypA knockdowns did not lead to meaningful reductions in lifespan at these lower concentrations, suggesting that these genes may be less critical for aphid survival under the conditions tested, or that functional redundancy may compensate for their loss. eIF2B emerged as a particularly promising target, as its knockdown significantly reduced aphid survival even at lower dsRNA concentrations as shown in Figures 4–6 [33,34].

Building on these findings, future research should focus on expanding the range of candidate genes within the UPR. Another important direction for future work is the development of transgenic plants capable of expressing dsRNA targeting essential aphid genes. By incorporating RNAi constructs into crop genomes, it may be possible to control aphid infestations this way, reducing reliance on traditional chemical insecticides. This approach not only has the potential to improve agricultural sustainability but also minimizes environmental impact and the risk of off-target effects associated with chemical treatments [35,36].

In addition to single gene targeting, combining RNAi treatments should be explored to assess whether simultaneous knockdowns of multiple genes produce synergistic effects. Targeting multiple components of the UPR may enhance overall effectiveness and reduce the likelihood of resistance development. These experiments could be conducted using similar methodologies to the single-gene assays described here.

## CONCLUSION

Overall, this study reinforces the potential of RNAi as a powerful tool for aphid control and highlights eIF2B as a strong candidate for further investigation. Continued research into gene targets, delivery systems and combination approaches will be essential for translating these findings into practical agricultural applications.

## Figures and Tables

**Table 1:** 1 ug/mL feeding study data.

Time (hrs)	Control	RBN1	eIF2B	CypA	Time (hrs)	Control

0	50	50	50	50	70	80
6	50	50	50	50	67.6	76.4
9	48	45	48	40	56.6	62.8
15	47	43	42	37	48.5	52.4
21	46	39	36	33	39.2	40.6
27	43	37	30	29	30.5	29.6
32	40	30	24	28	23.6	21.2
44	38	27	24	23	14.4	8.8
50	8	0	1	0	−20.3	−31
56	6	0	0	0	−23	−34.8
58	0	0	0	0	−23.2	−34.8

**Note:** Mentel-cox results; Control *vs* RBN1; P-value 0.001561412; Control *vs* eIF2B; P-value 0.0002611268; Control *vs* CypA; P-value 0.0001032172

**Table 2:** 100 ng/mL feeding study data.

Time (h)	Control	RBN1	eIF2B	CypA

0	50	50	50	50
6	46	48	38	40
12	48	46	38	40
18	36	33	27	27
24	23	19	16	12
28	4	7	3	9
34	4	4	0	1
46	4	0	0	0
50	4	0	0	0
51	3	0	0	0
52	3	0	0	0
53	3	0	0	0
57	2	0	0	0
59	2	0	0	0
73	2	0	0	0
78	1	0	0	0
82	0	0	0	0

**Note:** Mentel-cox results; Control *vs* RBN1; P-value 0.4243546; Control *vs* eIF2B; P-value 0.04484973; Control *vs* CypA; P-value 0.08461485

**Table 3:** 10 ng/mL feeding study data.

Time (h)	Control	eIf2B

0	50	50
4	48	45
10	44	42
16	42	39
22	41	37
27	39	34
30	34	29
34	31	26
40	19	15
46	5	4
52	0	0

**Note:** Mentel-Cox results; Control *vs* eIF2B; P-value 0.3133068

**Table 4:** Primer pairs of chosen genes.

	Forward	Reverse

RPN1	AGAAGCTGGATGCACAGGTC	AAACGGCAAACTCACACTGC
eIF2B	TAGGGATTGGCTACAGGGCT	AAATGTGAGCCATCCTGGGG
CypA	GTTCTTCGACATTGCCGTCG	CTCAGTCTTGGCAGTGCAGA

**Table 5:** Akey-beck diet.

Essential amino acids	

L-Arginine HCI	12.5 mM
L-Histidine	7.5 mM
L-Isolucine	7.5 mM
L-Leucine	7.5 mM
L-Lysine HCI	7.5 mM
L-Methionine	2.5 mM
L-Phenylalanine	2.5 mM
L-Threonine	7.5 mM
L-Tryptophan	2.5 mM
Nonessential amino acids	
L-Valine	7.5 mM
L-Alanine	5 mM
L-Asparagine	12.5 mM
L-Aspartic acid	12.5 mM
L-Cysteine HCI	2.5 mM
L-Cysteine	0.2 mM
L-Glutamic acid	7.5 mM
L-Glutamine	15 mM
Glycine	1 mM
L-Proline	5 mM
L-Serine	5 mM
L-Tyrosine	0.5 mM
Trace metals	
Gamma amino butyric acid	2 mM
Cupric chloride	14 μM
Ferric chloride	49 μM
Magnesium (II) chloride	40 μM
Zinc sulfate	30 μM
Salts, Buffers and sterol	
Calcium citrate	0.175 mM
Cholesterol benzoate	50 μM
Potasium phosphate	18.37 mM
Sodium chloride	0.127 mM
Magnesium chloride	9.837 mM
Vitamins	
Choline chloride	3.579 mM
p-Aminobenzoic acid	0.73 mM
Ascorbic acid	5.68 mM
D-Calcium pantothenate	0.21 mM
Folic acid	22 mM
Inositol (meso) dihydrate	1.39 μM
Nicotinc acid	0.812 mM
Pyridoxine HCI	0.21 mM
Thiamine HCI	74 μM
Sugars	
Sucrose	0.5 mM
